# Operationalizing insect resistance management for commercialized Bt crops: an evidence-to-action framework for post-market stewardship

**DOI:** 10.1080/21645698.2026.2709923

**Published:** 2026-07-27

**Authors:** Zhanfeng Yan, Dapeng Jing, Zhenying Wang

**Affiliations:** a State Key Laboratory of Crop Germplasm Innovation and Molecular Breeding, Syngenta Biotechnology (China) Co., Ltd., Beijing, China; b State Key Laboratory for Biology of Plant Diseases and Insect Pests, Institute of Plant Protection, Chinese Academy of Agricultural Sciences, Beijing, China

**Keywords:** Bt crops, evidence-to-action framework, insect resistance management, post-market stewardship, practical resistance, resistance monitoring

## Abstract

Commercialized *Bacillus thuringiensis* (Bt) crops require post-market stewardship that translates heterogeneous resistance signals into defensible action. Although previous syntheses have defined field-evolved and practical resistance and summarized global patterns, developers, regulators, and stewardship programs still need an operational structure for distinguishing weak anomalies from evidence warranting confirmation, mitigation, deployment revision, or replacement of compromised components. We synthesize evidence and stewardship experience from Bt maize and Bt cotton into an evidence-to-action framework linking preventive IRM implementation, resistance monitoring, evidence-state assignment, actionability assessment, and proportionate response. The framework classifies outcomes into four states: baseline susceptibility, early-warning signal, confirmed field-evolved resistance, and practical resistance. For each state, we define its operational meaning, minimum verification requirements, and proportionate actions. Representative pest – crop – toxin systems illustrate how field performance, bioassays, mechanistic evidence, deployment history, and refuge context can support earlier, more transparent, and evidence-proportionate stewardship decisions.

## Introduction: Bt Crop Commercialization Requires Post-Market Stewardship

*Bacillus thuringiensis* (Bt) maize and Bt cotton are among the most widely adopted genetically modified (GM) crop applications for insect pest management. These crops can suppress major lepidopteran pests, reduce reliance on broad-spectrum insecticides, and stabilize yield protection when deployed within effective insect resistance management (IRM) programs.^1,2^ Their long-term value, however, depends not only on the intrinsic activity of expressed proteins, but also on whether post-market stewardship can detect, interpret, and respond to resistance signals before efficacy loss becomes widespread.

The commercial phase of Bt crop use creates an evidence-to-action problem. Monitoring programs may detect unusual field injury, survival in diagnostic-dose assays, shifts in dose-response metrics, molecular-marker changes, or localized reports of control failure. Each signal may be meaningful, but each may also reflect confounders such as trait misidentification, uneven expression, pest-stage effects, sampling error, background variation in susceptibility, insecticide history, or poor refuge implementation.^3–6^ The practical question for stewardship is therefore not simply whether a pest population is resistant, but what level of evidence is sufficient to justify further investigation, mitigation, deployment revision, or replacement of a compromised trait package.

This question has become more important as Bt crop deployment has moved from single-toxin products toward pyramids, seed blends, and multi-crop landscapes in which the same pest populations may encounter overlapping Cry and Vip proteins across seasons and host crops. Pyramids can improve durability when each component remains effective and cross-resistance is limited, but they can also become selection traps when one component is already compromised or when deployment histories repeatedly expose pest populations to related toxin sets.^7–9^ As commercialization expands into production contexts with different refuge feasibility, farm structure, and monitoring capacity, IRM must be treated as a continuing stewardship process rather than as a static registration-stage requirement.

**Figure 1.**
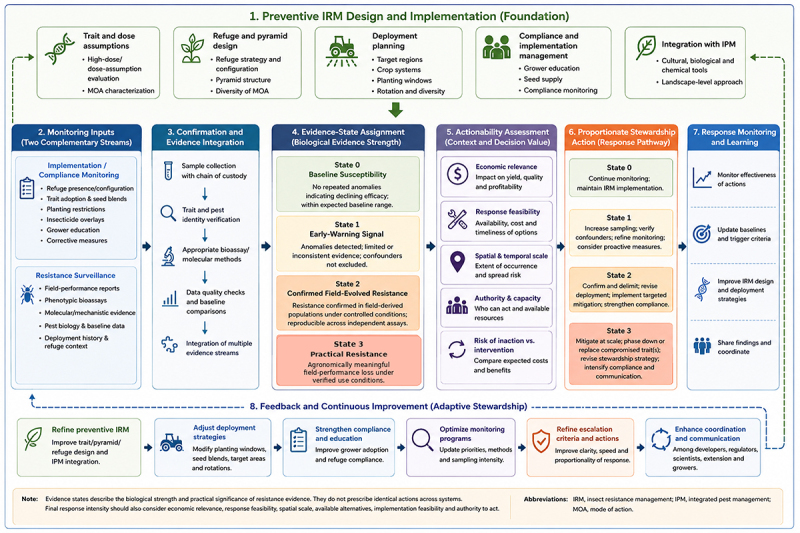
Integrated evidence-to-action framework for post-market stewardship of commercialized Bt crops. preventive IRM design and implementation, including trait and dose assumptions, refuge and pyramid design, deployment planning, compliance and implementation management, and integration with IPM, provide the foundation for post-market stewardship. Implementation/compliance monitoring and resistance surveillance generate two complementary evidence streams on whether the IRM plan is being followed and whether pest susceptibility or field performance is changing. Confirmation and evidence integration include sample handling and identity verification, appropriate phenotypic or molecular testing, data-quality checks, baseline comparisons, and integration of multiple evidence streams. The resulting evidence is assigned to one of four states: baseline susceptibility, early-warning signal, confirmed field-evolved resistance, or practical resistance. Practical resistance is characterized by agronomically meaningful field-performance loss under verified use conditions. Evidence-state assignment is followed by an actionability assessment that considers economic relevance, response feasibility, spatial and temporal scale, authority and capacity to act, and the risk of inaction versus intervention. Proportionate stewardship actions are then selected according to the evidence state and actionability context. Response monitoring and learning feed into continuous improvement of preventive IRM design, deployment strategies, compliance and education, monitoring priorities, escalation criteria, coordination, and communication.

Previous work has provided the conceptual foundation for this process. Field-evolved resistance and practical resistance have been clearly defined, early detection and mitigation have been analyzed in specific pest systems, and global patterns of Bt resistance have been reviewed across the first decades of commercial use.^3,10,11^ The remaining gap is operational: developers, regulators, and stewardship programs still need transparent criteria for assigning evidence strength and linking that evidence to proportionate action. Building on these foundations, this article develops an evidence-to-action framework for post-commercialization IRM in Bt crops. The framework organizes heterogeneous monitoring signals into evidence states and links each state to verification requirements and proportionate stewardship actions. The overall pathway linking preventive IRM design, implementation and resistance monitoring, confirmation, evidence-state assignment, actionability assessment, proportionate stewardship action, and post-action learning is summarized in Fig. 1.

## Positioning of This Article Relative to Previous Bt Resistance Syntheses

Established resistance terminology, global syntheses, and regulatory experience provide the conceptual foundation for this article. Previous studies have defined field-evolved resistance and practical resistance, summarized global resistance patterns, and examined early detection, mitigation, and governance of Bt crop resistance. Building on these foundations, the present article organizes heterogeneous post-market evidence into a four-state decision architecture that links monitoring outputs to verification requirements, actionability assessment, and proportionate stewardship responses. Recent work has also organized Bt resistance diagnostics into a mechanism-informed workflow linking phenotypic confirmation, breadth and cross-resistance mapping, mechanism inference, and assessment of marker readiness for surveillance.^12^ The present article operates at a complementary decision level by integrating field performance, phenotypic and mechanistic evidence, deployment and compliance context, and actionability considerations into post-market evidence states and proportionate stewardship responses.

The contribution is threefold. First, descriptive information from field-performance observations, phenotypic bioassays, molecular or mechanistic studies, and deployment history is converted into explicit evidence-state assignments. Second, each evidence state is paired with minimum verification requirements and a transparent account of remaining uncertainty. Third, biological evidence strength is separated from the subsequent assessment of economic relevance, response feasibility, and available management options. This structure provides a common decision language for developers, regulators, extension programs, and growers.

The framework also integrates established regulatory and stewardship practice. In the United States, Bt plant-incorporated protectant IRM requirements and Scientific Advisory Panel recommendations have long incorporated preventive trait and refuge strategies, resistance monitoring, compliance assessment, grower education, remedial action, annual reporting, and revision of IRM requirements as new evidence emerges.^13,14^ The relationship between the present framework and foundational Bt resistance literature and regulatory practice is summarized in Table 1.

**Table 1.** Relationship of the present article to foundational Bt resistance literature and regulatory practice.

Previous literature	Main contribution	Operational gap addressed in the present article
^10^	Defined key resistance terms, including field-evolved resistance and practical resistance.	Uses those definitions as endpoints in a practical evidence-to-action pathway rather than proposing new terminology.
^3^	Analyzed early detection and mitigation logic for resistance management.	Extends early-detection logic into a cross-system post-market stewardship matrix linking evidence, verification requirements, and action.
^11^	Summarized global patterns of insect resistance to transgenic Bt crops over the first 25 years.	Moves from global pattern description to operational assignment of evidence states for developers, regulators, and stewardship programs.
^15^and related socioecological work	Highlighted governance, compliance, and institutional dimensions of Bt crop resistance management.	Connects governance constraints to specific evidence states and proportionate stewardship action options.
US EPA Bt PIP IRM framework and SAP recommendations	Established regulatory expectations for refuge-based IRM, resistance monitoring, compliance, remedial action, and post-market stewardship of Bt crops.	The present article generalizes these regulatory concepts into a cross-system evidence-state framework for interpreting heterogeneous monitoring evidence and linking it to proportionate action.

Note: Although some foundational decision-making literature from non-lepidopteran Bt maize systems is cited to support the logic of early detection and mitigation, the case matrix below focuses primarily on representative lepidopteran Bt maize and Bt cotton systems because these provide the most directly comparable post-market evidence streams for the present framework.

## Review Approach and Evidence Classification

This article uses a targeted, evidence-informed synthesis to develop an operational decision framework and compare the stewardship implications of contrasting evidence packages. Bt maize and Bt cotton were selected because these systems provide the most extensive published experience with field-performance monitoring, diagnostic bioassays, practical resistance, refuge implementation, and post-market stewardship. The analysis focuses on major lepidopteran pest – crop – toxin systems that illustrate how evidence strength changes across the transition from baseline susceptibility to practical resistance.

Literature searches were last updated on 30 April 2026. A directly relevant review published during manuscript revision was subsequently included to clarify the relationship between mechanism-informed diagnostics and the present post-market stewardship framework. Studies were identified from Web of Science, Scopus, CAB Abstracts, and Google Scholar using combinations of terms related to Bt crops, maize, cotton, Cry proteins, Vip3Aa, resistance monitoring, field-evolved resistance, practical resistance, diagnostic dose, F2 screen, molecular marker, refuge, pyramid, seed blend, stewardship, and deployment. Cases were eligible for detailed consideration when they contained at least one stewardship-relevant evidence stream, including verified field-performance observations, replicated phenotypic bioassays, resistance-ratio or diagnostic-dose data, inheritance or fitness-cost analysis, molecular or mechanistic evidence, deployment or refuge information, or explicit discussion of stewardship action. Cases were selected to represent contrasting evidence packages and stewardship contexts across the transition from baseline susceptibility to practical resistance. This case-based structure allowed comparison of how field performance, phenotypic resistance, mechanistic evidence, and deployment context alter the appropriate stewardship response.

The resulting evidence classification is designed as an adaptable decision-support framework that can be calibrated to local monitoring capacity, pest biology, deployment history, baseline quality, and available management options. Four evidence states are used: baseline susceptibility, early-warning signal, confirmed field-evolved resistance, and practical resistance. These states correspond to different levels of verification and different default stewardship responses. Because the objective was framework development rather than quantitative synthesis, studies were interpreted for evidentiary diversity and stewardship relevance, without statistical pooling.

## Alignment with Existing Regulatory and Stewardship Frameworks

Existing national and regional Bt crop IRM programs differ in legal authority, preventive controls, monitoring design, and the strength of the link between monitoring evidence and remedial action. The US EPA system combines preventive IRM planning with compliance monitoring, resistance monitoring, unexpected-damage investigation, annual reporting, and remedial action. In the European Union, monitoring of MON810 is implemented through post-market environmental monitoring, including case-specific monitoring, general surveillance, refuge-compliance assessment, and annual scientific review. Australian Bt cotton stewardship combines regulatory oversight with industry-coordinated resistance management plans that include refuge crops, planting restrictions, pupae-busting cultivation, control of volunteer and ratoon cotton, and spray limitations. These experiences demonstrate that resistance monitoring is most effective when embedded within preventive implementation controls and predefined response pathways.^13,14,16–18^

Because the availability and formalization of public regulatory documentation differ among jurisdictions, Table 2 distinguishes formal regulatory programs from broader national implementation contexts. The comparison identifies common elements, major differences, and improvement priorities relevant to the present evidence-to-action framework.

**Table 2.** Alignment of the evidence-to-action framework with selected regulatory programs and Bt crop IRM implementation contexts.

Program or context	Preventive and implementation elements	Post-market evidence and response pathway	Main gap or improvement priority	Key references
United States – US EPA Bt PIP IRM	Dose/refuge strategy, product-use requirements, grower education, compliance monitoring, annual reporting, and preventive IRM planning	Resistance monitoring, unexpected-damage investigation, confirmatory evaluation, remedial action, annual reporting, and product-management or phase-down measures	Improve the speed and consistency of response to emerging resistance and strengthen coordination across crops, pyramids, and shared toxin sets	^13–15^
European Union – EFSA/MON810	Case-specific monitoring, general surveillance, refuge requirements, and annual post-market environmental monitoring review	Target-pest susceptibility monitoring, refuge-compliance assessment, annual scientific evaluation, and recommendations to risk managers	Improve sampling power in uneven-adoption landscapes, harmonize diagnostic methods, and focus monitoring in areas with the greatest selection pressure	^16,17,19^
Australia – Bt cotton RMP	Refuges, planting windows or restrictions, pupae-busting cultivation, volunteer and ratoon cotton control, spray limitations, and coordinated grower requirements	Industry- and regulator-coordinated resistance monitoring, compliance oversight, and periodic revision of resistance management plans	Maintain high compliance and continually update plans as trait combinations, pest complexes, and deployment patterns change	^15,18^
Brazil – implementation context	Refuge recommendations, industry stewardship, pyramided traits, and high Bt maize adoption	Field-performance reports, phenotypic testing, resistance confirmation, and deployment revision when compromised control components are identified	Address variable refuge compliance, repeated exposure to related toxin sets, cross-crop selection, and delayed mitigation of compromised components	^11,15,20^
India – implementation context	Bt cotton refuge and stewardship requirements implemented in a predominantly smallholder production system	Field-performance monitoring, resistance confirmation, and regional pest-management responses	Improve refuge implementation, seed-system accountability, regional coordination, and access to effective alternative control tools	^9,11,15,21^
China – implementation context	Natural-refuge contributions, susceptibility monitoring, resistance-allele studies, and evolving needs for post-market Bt crop stewardship	Laboratory and field monitoring combined with deployment history, landscape information, and emerging post-market stewardship planning	Develop scalable post-market monitoring, predefined escalation criteria, and clearer assignment of response responsibilities under broader Bt crop deployment	^9,11,15,22,23^

Note: United States, European Union, and Australia are presented as formal regulatory or coordinated stewardship programs. Brazil, India, and China are presented as broader implementation contexts because the availability and formalization of public regulatory documentation differ among jurisdictions.

Across these programs and implementation contexts, the common element is the use of post-market information to detect changes in pest susceptibility, field performance, or IRM implementation. The main differences lie in legal authority, compliance monitoring, field-damage investigation, and the extent to which monitoring outputs trigger predefined remedial actions. The principal improvement priorities are more consistent baseline quality, stronger implementation and compliance monitoring, clearer sample-routing procedures, predefined escalation criteria, and earlier identification of feasible actions once resistance evidence emerges.

## Bt Crop Stewardship as an Evidence-To-Action Problem

Bt crop IRM is often described through biological principles such as high-dose expression, refuge maintenance, pyramided toxins, and reduced cross-resistance.^1,7,8,24^ These principles remain essential, but during commercialization they must be translated into monitoring routines and action thresholds. A stewardship program needs to know which indicators are routinely collected, which anomalies trigger repeat sampling, how field and laboratory evidence are reconciled, who evaluates inconclusive evidence, and which mitigation options are feasible before widespread loss of control occurs.^3,15^ Effective IRM begins with preventive design and implementation, including appropriate trait and dose assumptions, refuge and pyramid design, deployment planning, compliance management, and integration with non-Bt IPM tools. Resistance monitoring is one component of this broader system and provides feedback on whether the preventive strategy continues to function as intended. Implementation monitoring evaluates whether the IRM plan is being adopted and followed, resistance monitoring evaluates biological changes in pest susceptibility or field performance, and response monitoring evaluates whether mitigation measures are implemented and effective. Accordingly, the evidence-to-action framework functions as a decision layer within a broader IRM system.

The same monitoring observation can have different meanings in different deployment contexts. Survival at a diagnostic concentration may be alarming in a system with a stable local baseline and validated assay conditions, but less interpretable in a system without a susceptible reference or reliable field metadata. Unexpected field injury may suggest practical resistance when it recurs under verified use conditions, but it may also reflect nonresistance causes such as incorrect hybrid identity, late planting, pest pressure outside the intended spectrum, or injury by a different pest species. Similarly, molecular evidence can strengthen interpretation when genotype-phenotype relationships are established, but it should not generally substitute for phenotypic confirmation when management escalation is being considered.

For this reason, stewardship decisions should be evidence-state decisions. The purpose is to make evidence, uncertainty, and proportionate action explicit. This framing reduces two opposite risks: dismissing repeated early-warning signals as background noise, and over-interpreting isolated anomalies as practical resistance.

## Evidence Streams for Post-Commercialization IRM

### Field-Performance Signals

Field-performance data are the most direct indicator of whether a Bt crop is providing acceptable pest control under production conditions. Relevant observations include injury severity, surviving larvae, spatial clustering of damage, trait identity, pest identity, crop stage, planting date, refuge context, insecticide applications, and whether similar damage recurs across fields or seasons. Field signals are essential for identifying practical consequences, but they are vulnerable to confounding and therefore require contextual verification before they are treated as resistance evidence.^4,25–27^ Therefore, field-performance signals should normally be treated as triggers for investigation rather than as stand-alone proof of resistance unless they are repeated, verified, and supported by phenotypic confirmation.

### Phenotypic Bioassays

Phenotypic bioassays remain the primary route for converting field suspicion into comparable biological evidence. Diagnostic-dose assays are useful for rapid surveillance relative to a baseline, whereas dose-response assays provide stronger confirmation when a warning signal requires follow-up.^28–30^ Interpretability depends on validated protocols, adequate sample sizes, appropriate controls, resistance-ratio estimates, confidence intervals, and documentation of toxin source and assay conditions. For escalation decisions, repeated phenotypic evidence from independent collections is usually more persuasive than a single anomalous assay result.

### Molecular and Mechanistic Evidence

Mechanistic evidence can sharpen diagnosis and inform cross-resistance expectations. Examples include cadherin, tetraspanin, ABC transporter, altered toxin binding, and chitin synthase loci associated with resistance to Cry or Vip proteins in specific systems.^31–34^ Marker evidence is strongest when genotype-phenotype relationships are validated in the relevant pest and region. Because resistance can be polygenic, mechanism-specific, or context-dependent, marker evidence should usually complement phenotypic confirmation, particularly when regulatory escalation, product restriction, or major deployment revision is being considered.

### Deployment History and Refuge Context

Deployment history determines how monitoring signals should be interpreted. Sequential exposure to related proteins, widespread adoption of a trait package, poor refuge compliance, and movement across maize-cotton or multi-host landscapes can increase selection pressure and reduce the durability expected from pyramids.^8,9,20^ Conversely, abundant effective refuges, diversified host landscapes, and coordinated stewardship can slow resistance evolution and help preserve susceptibility.^15,22,23^

### Compliance and Adoption Monitoring

Compliance and adoption monitoring provide direct information on whether the intended IRM strategy is being implemented. Relevant indicators include refuge planting, refuge configuration, seed-blend use, trait adoption rate, repeated use of the same toxin set, grower education, insecticide overlay, and adherence to any planting restrictions or remedial action requirements. These data are different from pest susceptibility data, but they strongly affect how susceptibility signals should be interpreted. For example, a susceptibility shift in a region with poor refuge compliance and high adoption of a single toxin set may justify more rapid escalation than the same shift in a region with diversified deployment and strong compliance. Conversely, where compliance monitoring shows that the IRM plan has not been implemented, the first stewardship response may need to address implementation failure rather than infer biological resistance from field injury alone.

Compliance and adoption monitoring should therefore be treated as an independent evidence stream within post-market stewardship. It helps distinguish biological resistance from implementation failure and may indicate that corrective action should first target refuge compliance, deployment practices, or grower communication before resistance is inferred from field injury alone.^13,15,18^

## Evidence-To-Action States for Bt Crop Stewardship

The four-state framework below is adapted for stewardship decision-making. It preserves the established distinction between field-evolved resistance and practical resistance, but adds explicit attention to verification requirements, interpretive uncertainty, and proportionate stewardship action. These four states, their operational definitions, verification requirements, and default stewardship actions are summarized in Table 3.

**Table 3.** Evidence-to-action states for post-commercialization Bt crop stewardship.

State	Operational definition	Minimum verification requirements	Proportionate stewardship action
State 0. Baseline susceptibility	Bioassay response and field performance remain within the expected local range defined by historical baseline data, susceptible references, or validated local monitoring experience. No repeated anomalies indicating declining efficacy are observed	Routine surveillance with standardized protocols, valid controls, trait and pest identity checks, and documented field metadata	Maintain routine stewardship, baseline updating, refuge communication, and compliance review
State 1. Early-warning signal	Repeatable but still unconfirmed evidence suggests declining susceptibility or abnormal performance	Repeat sampling from independent fields or populations; verify trait and pest identity; repeat diagnostic-dose or dose-response assays; review deployment and refuge context	Increase sampling intensity, initiate confirmatory testing, review exposure history, and consider low-risk mitigation such as refuge-compliance review, grower communication, and targeted IPM support where feasible
State 2. Confirmed field-evolved resistance	Replicated phenotypic evidence from field-exposed populations consistently indicates a heritable or likely genetically based reduction in susceptibility, even if repeated field efficacy loss has not yet been demonstrated	Consistent phenotypic signal across independent collections or assays; major confounders reviewed and excluded as far as practical; inheritance tests, F2 screens, laboratory selection, resistance-allele estimates, or validated marker evidence used where feasible	Implement targeted mitigation: tighten refuge and compliance measures, avoid repeated exposure to the affected toxin set, intensify targeted IPM, update monitoring priorities, and revise deployment plans where feasible
State 3. Practical resistance	Confirmed field-evolved resistance accompanied by repeated and agronomically meaningful loss of Bt crop efficacy under verified use conditions	Documented field-performance shortfall under correct trait identity and recommended use conditions, together with phenotypic confirmation from affected populations across more than one independent site, sampling round, or season unless failure is severe, widespread, and supported by strong confirmatory evidence	Replace or restrict the compromised control component where feasible, coordinate response across the affected production area based on pest movement and deployment context, deploy effective alternatives, and evaluate post-action outcomes

## Case-Based Evidence Matrix Across Bt Maize and Bt Cotton Systems

Published cases differ in the strength and type of evidence available. Some systems illustrate early warning without documented practical failure, whereas others combine field-performance loss with phenotypic confirmation and therefore justify stronger action. Table 4 provides a representative evidence matrix showing how selected pest – crop – toxin systems can be mapped to the four-state framework.

**Table 4.** Representative Bt crop resistance and stewardship cases mapped to evidence-to-action states.

Pest-crop-toxin system	Evidence profile	Evidence convergence	Evidence-state interpretation in this framework	Stewardship lesson
*Helicoverpa armigera* - Bt cotton - China - Cry1Ac	Repeated laboratory evidence of reduced susceptibility and mechanism-informed signals; field-control failure was not the central basis for interpretation.^11,29,31^	Phenotype + mechanism; limited documented practical field failure	State 1 under warning-only conditions; State 2 only where local confirmation shows replicated phenotypic resistance	Treat as warning or early escalation: maintain baseline monitoring, verify recurrence, and intensify confirmation before claiming practical resistance.
*Pectinophora gossypiella* - Bt cotton - China - Cry1Ac	Reported susceptibility shifts in monitoring studies without the same practical failure pattern observed elsewhere.^9,11,29^	Laboratory susceptibility shift; limited documented practical field failure	State 1	Use as early-warning evidence; avoid over-interpreting laboratory shifts without agronomic failure.
*P. gossypiella* - Bt cotton - India - Cry1Ac and Cry1Ac+Cry2Ab	Multiple studies and field observations document resistance and substantial field damage after prolonged deployment.^9,21,35,36^	Field failure + phenotype + prolonged deployment history	State 3	Practical resistance supports replacement, stronger refuge and IPM, and region-level response.
*P. gossypiella* - Bt cotton - United States - Cry1Ac	Long-term monitoring and eradication/stewardship programs preserved control and supported regional suppression.^9,37^	Stewardship-success comparator; monitoring + coordinated suppression	State 0 / stewardship-success comparator	Shows that coordinated stewardship, monitoring, and compliance can preserve Bt crop utility; this row is a comparator rather than a resistance-failure case.
*Spodoptera frugiperda* - Bt maize - Puerto Rico - Cry1F	Unexpected field damage plus phenotypic characterization established resistance and practical performance loss.^25,38^	Field failure + phenotypic confirmation + product-use history	State 3	Product restriction or withdrawal and substitution with effective alternatives are justified.
*S. frugiperda* - Bt maize - Brazil - Cry1F and Cry1Ab	Field-evolved resistance documented under sustained selection pressure; reduced efficacy occurred in commercial systems.^39,40^	Phenotype + reduced field performance + sustained deployment pressure	State 3	Illustrates how sequential deployment of compromised traits can accelerate loss of control.
*S. frugiperda* - Bt maize - Brazil - Cry1A.105+Cry2Ab and Cry1F	Practical resistance of two Brazilian populations to Cry1A.105+Cry2Ab and Cry1F maize was documented with high survival and life-table evidence.^20^	Field performance + high survival + life-table evidence + prior exposure	State 3	Shows that pyramiding alone does not guarantee durability when resistance alleles, prior exposure, and cross-resistance risks are present.
*S. frugiperda* - Bt maize - Colombia - Cry1A.105+Cry2Ab2+Cry3Bb1	Control failures in commercial fields, survival on pyramided maize, and retained susceptibility to Vip3Aa20; Cry3Bb1 is part of the trait package but is not the primary FAW-active component.^27^	Field failure + survival on FAW-active Cry1/Cry2 components + Vip3Aa susceptibility contrast	State 3 for the FAW-active Cry1/Cry2 component; State 0/1 for Vip3Aa20 in tested populations	Supports deployment revision while emphasizing the need to preserve Vip3Aa susceptibility.
*Helicoverpa zea* - sweet corn/maize/cotton - United States - Cry1/Cry2 proteins	Repeated field efficacy loss and bioassay evidence indicate widespread resistance to several Cry proteins.^4,5,26,41^	Repeated field efficacy loss + phenotypic evidence + multi-crop deployment	State 3 for affected Cry proteins	Continued reliance on Cry-only control is not defensible; stewardship must prioritize Vip3Aa durability and IPM integration.
*H. zea* - maize/cotton - United States - Vip3Aa	F2 screens, resistance-allele estimates, reduced-binding evidence, laboratory-selected resistance, gene-editing evidence, and cross-resistance work provide early warning; broad field efficacy has generally remained better than for Cry-only control.^30,32,34,42–45^	F2 screens + resistant strains + binding/gene evidence; limited broad practical field failure	State 1 overall; State 2 only for local populations with replicated resistant phenotypes; not broad practical resistance	Use proactive monitoring, resistance-gene surveillance, and cross-resistance testing; avoid waiting for widespread Vip3Aa field failure before stewardship escalation.
*Busseola fusca* - Bt maize - South Africa - Cry1Ab	Field damage and laboratory confirmation documented resistance to Cry1Ab maize after commercial deployment.^11,46,47^	Field damage + laboratory confirmation + post-release deployment context	State 3	Illustrates the need for pest-specific dose assumptions, local refuge feasibility checks, and post-release monitoring.
*B. fusca* - Bt maize - South Africa - Cry2Ab2 in MON 89,034	Recent work reported resistance to Cry2Ab2 in populations collected from localities with greater-than-expected damage to MON 89,034.^48^	Phenotypic resistance + greater-than-expected field damage; practical failure requires local verification	State 2; State 3 only where local field-performance loss is repeatedly verified under correct trait use conditions	Second-generation Bt maize can also become vulnerable; monitoring must continue after product replacement or trait switching, and local field-performance verification is needed before assigning practical resistance.
*Ostrinia nubilalis* - Bt maize - Canada/United States - Cry proteins	Monitoring studies and marker work show how structured surveillance and mechanism-informed diagnostics can support early detection.^33,49^	Routine monitoring + diagnostic/marker workflow; no broad practical failure	State 0 under most routine monitoring contexts; State 1/2 only where validated diagnostic or marker evidence supports escalation	Demonstrates value of baseline preservation and validated diagnostic workflows.
*Sesamia nonagrioides* - Bt maize - European Union - MON810/Cry1Ab	Long-term harmonized monitoring of MON810 maize in the EU reported no major shifts in susceptibility to Cry1Ab and no signs of field resistance, while identifying practical constraints and recommendations for improving monitoring design.^19^	Long-term monitoring + no field resistance signal + technical reassessment of monitoring plan	State 0 / long-term monitoring comparator	Demonstrates the value of harmonized monitoring, reference strains or diagnostic concentrations, and focusing effort in high-adoption regions where selection pressure is greatest.

Note: State assignments in Table 4 are evidence-state interpretations based on published information rather than fixed global classifications. They should be updated when local field-performance verification, replicated phenotypic confirmation, validated marker evidence, or changes in deployment context become available. Evidence states describe the biological strength and practical significance of resistance evidence. They do not prescribe identical actions across all systems. Final response intensity should also consider economic relevance, availability of effective alternatives, expected spatial spread, implementation feasibility, crop value, and the authority of the responsible organization to act.

Case assignments in Table 4 were based on convergence among four evidence streams: verified field-performance loss, replicated phenotypic resistance, molecular or mechanistic support, and deployment/refuge context. State 3 was assigned only when agronomically meaningful field-control failure and phenotypic confirmation converged. State 2 was assigned when replicated phenotypic evidence supported field-evolved resistance but repeated practical field failure was not consistently documented. State 1 was assigned when warning signals were repeatable but still insufficient for confirmed field-evolved resistance, and State 0 was assigned when available evidence supported continued susceptibility or successful stewardship.

The matrix provides illustrative, decision-oriented examples selected to compare evidence packages with different stewardship implications. Across the matrix, practical resistance is most defensible when field-performance loss and phenotypic confirmation converge, as in several *S. frugiperda* and *H. zea* systems. In contrast, Vip3Aa-related evidence in *H. zea* should be interpreted as an early stewardship signal rather than evidence of broad practical resistance: allele-frequency estimates, resistant strains, reduced binding, gene-editing evidence, and cross-resistance analyses can justify intensified monitoring and deployment caution without being equated automatically with practical resistance.^32,34,42–45^

## Operational Trigger Criteria and Minimum Verification Requirements

Table 5 summarizes the minimum data elements needed to move from an initial anomaly to an evidence-state assignment. Trigger strength depends on the completeness and quality of the monitoring record. A field-injury report lacking verified trait identity, pest identity, crop stage, refuge context, or sample-routing information remains a weak signal because major confounders cannot be excluded. Repeated injury in verified Bt fields, linked to diagnostic-dose survival or a reproducible dose-response shift in field-derived insects, provides a stronger basis for confirmatory testing and targeted mitigation.

**Table 5.** Minimum data elements for an evidence-to-action monitoring pipeline.

Data element	Minimum fields	Quality checks	Decision value
Sampling/event metadata	Event ID, date, location, crop, pest species and life stage, collector.	Chain of custody, standardized pest identification, geospatial precision.	Supports repeat sampling and hotspot mapping.
Trait and exposure context	Bt event, expressed proteins, planting date, refuge type, insecticide history, agronomic notes.	Trait verification where feasible; refuge and MOA coding.	Helps separate resistance from deployment or management confounders.
Field-performance signal	Injury metrics, survival, pest density, spatial clustering, crop stage	Predefined rating scales and replication	Primary evidence for practical consequences
Bioassay metadata/results	Assay type, toxin source, diagnostic dose or dose series, controls, sample size, LC50/EC50 or survival.	Control mortality threshold, reference strain, confidence intervals, protocol version	Primary evidence for phenotypic confirmation
Mechanism/marker data	Target gene or marker, genotype counts or allele frequency, assay method	Validated genotype-phenotype link and laboratory QC	Strengthens interpretation and cross-resistance inference.
Response documentation	Assigned evidence state, action taken, implementation date, follow-up outcome	Time-stamped decision log	Enables post-action evaluation and transparent stewardship
IRM implementation and compliance	Refuge presence and configuration, trait adoption, seed-blend use, planting restrictions, grower education, insecticide overlays, and corrective measures	Defined audit method, representative coverage, documented source of compliance information, and consistency across reporting periods	Distinguishes biological resistance from implementation failure and identifies corrective stewardship actions

Note: “Chain of custody” refers to documented handling and transfer of field samples from collection to laboratory testing, including sample identity, storage, transport, and receipt records. “Time-stamped decision log” refers to a dated record of the evidence reviewed, evidence-state assignment, decision rationale, stewardship action taken or deferred, and follow-up outcome. “MOA coding” refers to classification of expressed Bt proteins or other control tools by mode of action to support interpretation of repeated exposure and cross-resistance risk.

Each trigger criterion should specify the observed anomaly, the comparison baseline, the minimum confirmation requirement, and the default next step. Numerical thresholds should be calibrated to local assay variance, pest biology, deployment history, and the relative costs of false alarms and missed detection. For example, a single unexpected-injury report may trigger sample collection and trait verification, whereas repeated injury in verified Bt fields combined with diagnostic-dose survival would justify confirmatory dose-response testing and consideration of targeted mitigation.

For field-performance signals, escalation is strongest when injury or survival recurs under verified use conditions and cannot be explained by trait misidentification, pest misidentification, crop stage, application history, or abnormal pest pressure. One damaged field should normally trigger investigation; repeated damaged fields with correct trait identity and phenotypic confirmation can support practical resistance assignment, as illustrated by documented cases in *S. frugiperda* and *H. zea*.^4,25–27^ Unexpected injury should therefore be defined relative to the labeled target spectrum, verified trait identity, crop stage, pest species and life stage, local injury history, and comparable non-Bt or alternative-trait checks where available.

For phenotypic assay signals, early warning is most credible when diagnostic-dose survival or dose-response shifts exceed local expectations and recur in independent collections. Resistance ratios should be interpreted relative to assay variance, the susceptible reference, toxin source, and whether the affected protein is expected to function as high dose against the target pest and tissue.^29,30,42,43^

For molecular-marker signals, escalation depends on the strength of the genotype-phenotype relationship. Marker frequency changes may justify targeted sampling and phenotyping when mechanisms are validated, but marker shifts alone should not generally be treated as practical resistance. The strongest case arises when markers, bioassays, and field-performance observations all point in the same direction.^31–34^

Sampling design is therefore a stewardship decision. When the objective is to detect rare events, simple binomial planning can provide a transparent starting point. Detecting at least one target event with 95% probability requires approximately 29 individuals if the target frequency is 10%, 59 if it is 5%, 149 if it is 2%, and 299 if it is 1%. These values assume independent sampling and should be treated as planning guides rather than substitutes for stratified field sampling; sample sizes should be adjusted upward when larvae are spatially clustered, collected from related egg masses, drawn from a small number of fields, or exposed unevenly across the landscape.

## Actionability, Economic Relevance, and Feasibility of Response

Before a resistance-monitoring program is initiated, its management objective, decision options, economic relevance, and feasible response measures should be defined. Monitoring has limited stewardship value when its results cannot alter a practical management or deployment decision. The choice between proactive and reactive monitoring should therefore reflect the target pest, expected resistance risk, economic importance of the trait, available mitigation options, and the time required to implement an effective response.^50^ Resistance monitoring is most valuable when the resulting data are actionable. A monitoring signal should therefore be interpreted not only by biological evidence strength, but also by economic relevance, feasibility of response, availability of effective alternatives, and the spatial scale over which action can be implemented. A confirmed susceptibility shift with little expected field or economic impact may justify continued observation or targeted confirmation rather than immediate high-cost intervention. Conversely, a moderate biological signal in a high-value crop, a high-adoption region, or a system with limited alternative control tools may justify earlier escalation.

Practical resistance also varies in stewardship significance. Some cases may remain localized, affect a minor pest, or occur in contexts where the affected toxin is no longer central to pest management. Other cases may threaten a widely deployed component, create cross-crop selection pressure, or compromise the durability of a pyramid. Therefore, evidence-state assignment should be followed by an actionability assessment that identifies the available decision, the organization with authority to act, the availability of effective alternatives, the expected cost of inaction, and the feasibility and cost of intervention.

This actionability step prevents two errors. The first is over-response to a technically interesting but low-impact signal. The second is delayed response to a biologically modest signal that is strategically important because it threatens a key control component or an entire deployment system. Thus, the framework should be used as a decision-support tool, not as an automatic trigger for identical mitigation in all cases.

## Implications for GM Crop Developers, Regulators, and Stewardship Programs

### For Technology Developers

Developers should treat IRM as a product-lifecycle responsibility. Pre-commercial efficacy and dose data are necessary but insufficient; products also need post-market surveillance plans that specify baseline datasets, diagnostic protocols, sample-routing pathways, and action thresholds. For pyramids, developers should document the expected contribution of each active component and update durability assumptions when evidence suggests that one component has become compromised.^8,9,45^ Where feasible, monitoring reports should distinguish whether reduced performance is associated with one active component, a shared mode-of-action group, or the full trait package, because these distinctions affect deployment revision and replacement options.

### For Regulators

Regulators can improve stewardship by requiring evidence-state reporting rather than only descriptive monitoring summaries. A practical reporting format could include the assigned evidence state for each monitored pest-crop-toxin system, the evidence supporting that assignment, unresolved uncertainty, changes from the previous reporting period, and the action taken or deferred. A monitoring report that states whether evidence remains at baseline, has moved to early warning, supports confirmed field-evolved resistance, or indicates practical resistance is more useful than a report that lists isolated observations without decision interpretation. Regulatory review should also encourage transparency about uncertainty, including when evidence is insufficient for escalation but sufficient to justify targeted confirmation.^3,15^

Regulators may also distinguish three monitoring layers: implementation monitoring, resistance monitoring, and response monitoring. Implementation monitoring asks whether the IRM plan is being adopted and complied with. Resistance monitoring asks whether pest susceptibility or field performance is changing. Response monitoring asks whether mitigation actions taken after an evidence-state change are implemented and effective. Separating these layers can help regulators determine whether a problem reflects biological resistance, poor implementation, inadequate monitoring design, or insufficient remedial action.

### For Stewardship and Extension Programs

Stewardship programs need practical communication procedures. Growers and field teams should know which field symptoms require reporting, what metadata must accompany samples, and how short-term IPM measures should be used while resistance confirmation is underway. In smallholder or capacity-limited systems, simplified evidence pathways may be more realistic than complex audit-based approaches, but simplification should not mean ignoring verification. The core requirement is a documented route from signal detection to confirmation and proportionate stewardship action.^15,36,51^

### For Regions Entering Expanded Bt Crop Deployment

Regions with expanding Bt crop commercialization can learn from earlier deployment histories. Baselines should be built before or during early commercialization; refuge feasibility should be evaluated under local production practices; pest movement and cross-crop exposure should be mapped where possible; and stewardship responsibilities should be assigned before evidence becomes contentious. Experience from China, India, Brazil, the United States, and South Africa shows that resistance outcomes depend as much on deployment context and follow-up capacity as on trait design alone.^11,20–23,48^ The highest-value action is to prevent ambiguous early-warning signals from becoming disputed only after widespread practical resistance has occurred. For regions where commercial Bt crop deployment is expanding after a long period of limited adoption, the most important early investment is not only efficacy testing, but also the creation of a pre-resistance decision infrastructure: baseline susceptibility data, sample-routing procedures, diagnostic bioassay capacity, refuge feasibility assessment, and predefined escalation criteria.

## Limitations and Research Needs

Because published cases differ in the type, quality, and completeness of available evidence, evidence-state assignment necessarily involves expert judgment; the framework is intended to make that judgment explicit rather than to eliminate uncertainty. The framework proposed here is intentionally operational and therefore cannot replace system-specific risk assessment. Evidence-state assignment depends on local baseline quality, available susceptible references, pest biology, field-sampling design, trait expression, and the feasibility of mitigation options. Some systems will require more conservative thresholds because effective alternatives are limited or because resistance is likely to spread rapidly. Other systems may tolerate more uncertainty before high-cost interventions are justified.

Future research should test whether evidence-state frameworks improve real stewardship outcomes. Useful evaluations would ask whether explicit evidence states shorten response time, improve consistency among technical reviewers, reduce disputes about inconclusive signals, or preserve trait efficacy longer than purely descriptive monitoring. Further research is also needed on Vip3Aa resistance mechanisms, Cry/Vip cross-resistance, practical sampling designs for low-frequency resistance alleles, and implementation models for fragmented or smallholder production systems.^32,34,42–45^

A second limitation is that many published cases contain uneven evidence. Some provide strong phenotypic and field-performance data but limited mechanism, whereas others provide laboratory or marker evidence without equivalent agronomic follow-up. This unevenness is precisely why evidence-state reporting is useful: it separates what has been confirmed from what remains uncertain and prevents either under-response to strong signals or over-response to isolated anomalies.

Finally, the framework does not imply that all evidence-state changes require identical actions across systems. Economic impact, crop value, availability of alternatives, regulatory authority, grower feasibility, and regional pest movement determine whether a technically confirmed signal is actionable and what level of response is proportionate.

## Conclusions

Bt crop durability depends on the quality of post-market stewardship as much as on the initial design of traits, refuges, and pyramids. Established resistance terminology distinguishes field-evolved resistance from practical resistance, but stewardship programs also need an operational pathway for interpreting earlier and weaker signals. The evidence-to-action framework proposed here links monitoring signals, verification requirements, and proportionate stewardship action across four evidence states: baseline susceptibility, early-warning signal, confirmed field-evolved resistance, and practical resistance. By making evidence strength and action requirements explicit, this framework can help developers, regulators, and stewardship programs respond earlier, communicate uncertainty more transparently, and preserve effective Bt crop technologies for longer. Its main value lies in enabling earlier, more transparent, and evidence-proportionate post-market decisions and in providing a common language for developers, regulators, extension programs, and growers.

## Data Availability

No new experimental data were generated for this article. The evidence summarized in the framework tables and case matrix is derived from the cited literature and should be updated as new resistance reports become available.
